# Modification of Macromolecules of Polyimide Films by Electron Irradiation

**DOI:** 10.3390/polym15092223

**Published:** 2023-05-08

**Authors:** Roza Abdulkarimova, Abyl Muradov, Kanat Mukashev, Gulmira Yar-Mukhamedova, Nursultan Japashov

**Affiliations:** 1 Institute of Experimental and Theoretical Physics, Al-Farabi Kazakh National University, 71, Al-Farabi Ave., Almaty 050040, Kazakhstan; abdulkarimovaroza@mail.ru (R.A.);; 2 Research Centre “KazAlfaTech LTD”, Karasu Str., 41A, Almaty 050020, Kazakhstan; 3 Faculty of Education and Humanities, Suleyman Demirel University, Almaty 040900, Kazakhstan

**Keywords:** polyimide, films, electron irradiation, modification of macromolecules, Infrared spectroscopy, electron paramagnetic resonance

## Abstract

New polymeric materials formation by controlling their properties is the primary and most challenging problem in developing a methodology for synthesizing a chosen technology and its use. The combined effect of high-energy electron radiation and tensile stress will cause a decrease in crystallinity and the breakage of chemical bonds in polyimide film macromolecules and is a new approach in their production technology. The effect of uniaxial tension and electron irradiation on the modification of polyimide film at room temperature was studied. Irradiation of the films caused an increase in the intensity of the IR spectrum by ~2–6 times and an increase in the width of the bands. The intensity in the range of 1700–3500 cm^−1^ increased, indicating an increase in the content of radicals as a result of irradiation. The amplitudes of the electron paramagnetic resonance signal from non-irradiated films increased from 3 × 10^3^ to 5 × 10^3^ as a result of uniaxial tension to fracture, indicating an increase in radicals in the material. The lines of the electron paramagnetic resonance spectrum shifted from 3475.0 cm^−1^ to 3512.5 cm^−1^, with a simultaneous decrease in the signal’s amplitude from 6 ×10^3^ to 4 × 10^3^, as a result of the electron irradiation of the films, followed by their subjection to tension. This was due to a decrease in the concentration of the radicals of the =N-H and –N-H_2_ groups until their disappearance and the formation of new ones.

## 1. Introduction

The intensive introduction of new polymeric and composite materials in technology and industry poses the problem of finding ways to develop them, so detailed studies are needed on their composition and structure, as well as the physicochemical processes occurring in each case. The physicochemical properties of polymeric materials are due to their specific structure in the form of a large number of repeating structural units interconnected by covalent chemical bonds [1,2,3,4]. Polyimides are widely used in the production of semiconductor devices. Among other polymers, they are distinguished by their characteristic physical properties such as high-temperature degradation, mechanical strength, and radiation resistance [5,6]. Polyimides have a low dielectric constant, which enhances signal propagation in electronic devices. Therefore, they are used in multilevel high-density and low-speed electronic circuits [7,8]. In the design of devices exposed to radiation, the properties of polyimides are stable during operation, so they are widely used [8,9,10,11].

Polyimides are ordered segments of the macromolecular chains of imide groups of the aromatic series. The degree of crystallinity of the polymer lies in the particular ordering of these chains at the molecular level. The amorphous phase in the material is a collection of disordered regions of macromolecules. Such structural features affect the various properties of the polymer, which significantly depend on the degree of order and their level of manifestation. Polymeric materials are characterized by the fact that their properties strongly depend on both the structure of the polymer chain and the mobility of the segments of individual sections of macromolecular chains.

At present, physical modification is widely used to change the physical properties of polymers, which consists of transforming the supramolecular structure by external physical influences without changing their chemical structure. Studies of the structural transformations of the modified polyimide, as well as studies of the physicochemical, mechanical, and operational properties of the newly obtained products, have not been deeply studied so far and are of practical interest. Conducting such studies is motivated primarily by practical interest in solving the problems of improving the reliability of instruments and devices operating in radiation fields and, secondly, by prospects for the development of radiation technologies that provide a directed modification of the physicochemical properties of materials.

The purpose of this work was to determine the features of the modification of macromolecules of polyimide films by changing the defects’ structure by irradiation with high-energy electrons and uniaxial tension.

## 2. Materials and Methods

Non-oriented polyimide (PI) films were chosen as the objects of this study. The choice of this material was based on the fact that it has a number of remarkable properties: it is a highly heat-resistant and fire-resistant material, it is resistant to radiation exposure and the action of weak acids, and also has good electrical insulating properties [7]. In addition to these factors, it also has good mechanical characteristics, such as the ability to maintain its strength across a wide temperature range.

**Figure 1:**
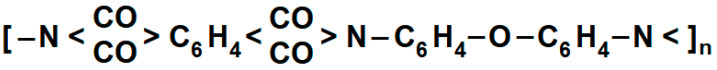
View of the structural unit of the polyimide macromolecule.

Polyimides belong to the class of heat-resistant synthetic polymers, the macromoleculare structure of which contains an imide cyclic group. The structural unit of a polyimide macromolecule based on pyromelitic dianhydride acid and paraoxydiphenylenediamine is shown in Figure 1 [7].

We made samples from polyimide films in the working part 5 cm long, 5 cm wide, and 130 µm thick. The prepared samples were radiated on a linear electron accelerator of the ELA-6 type. Radiation was provided by electron beams with an average energy of 2 MeV, with an integral current of up to 1000 µA, a pulse frequency of 200 Hz, and a duration of 5 µs. The doses (D) absorbed by the samples were 50 kGy, 500 kGy, and 40 MGy. Infrared (IR) spectroscopy was used to study the destruction of polyimides and analyze the products of chemical transformation after electron radiation. We used a Nicolet 5700 IR spectrometer (Thermo Electron Scientific Instruments Corp., Madison, WI, USA, 2019), which measured the spectra in the range of 4000–400 cm^−1^. The line power was 200 mWt with a spectral resolution of 0.09 cm^−1^.

In polymers, as a result of radiation exposure, compounds with free radicals are formed that have unpaired electrons, the electron paramagnetic resonance (EPR) study of which is very effective. The study of the nature of such a splitting of the spectrum, the number of lines, and their relative intensity made it possible to obtain an idea of the structure of a paramagnetic system and valuable information on its microdynamics. The EPR method relies on the fact that the frequency of scattered light depends on the strength of the bonds within the molecule, on the mass of the bound atoms, and on the intermolecular interaction. It provides valuable information about the structure of paramagnetic systems.

We used the Bruker ESP 300 E (Agilent Technologies, Santa Clara, CA, USA, 2020) spectrometer at a wavelength of 3.2 cm with a cavity power of ≤200 mWt, a modulation frequency of 100 kHz, and a sensitivity of 7 × 10^13^ sp/Tl. The magnetic field’s strength was 0.01–1.5 Tl, with a field stability of 10^−5^ per hour and a field uniformity of 10^−5^ Tl/cm. The central magnetic field had 34.70 G and a magnetic field sweep of 100 G. Two types of resonators were used: one rectangular (type TE-102) and cylindrical (type TE-011). The fabricated specimens were subjected to uniaxial tension to break at room temperature on an Instron 5982 tensile testing machine, which met all the requirements of both European and American standards. The Instron 5982 electromechanical machine had the following technical characteristics: the maximum allowable load was 100 kN, and the load and strain measurement errors were ±0.5% of the measured value [12].

In [13,14], regularities were established, and it was shown that the presence of certain functional groups in the macromolecules of organic compounds led to the appearance of radiation absorption with a characteristic frequency. Such spectra made it possible, with a high degree of probability, to identify whether one particular absorption band belonged to one particular functional group present in the molecule. The vibrations of the rest of the molecule were no more than 5% and did not affect the nature of the frequencies of the functional groups. Therefore, the obtained spectra of functional groups made it possible to accurately determine the structural features of the molecules of complex compounds.

**Figure 2:**
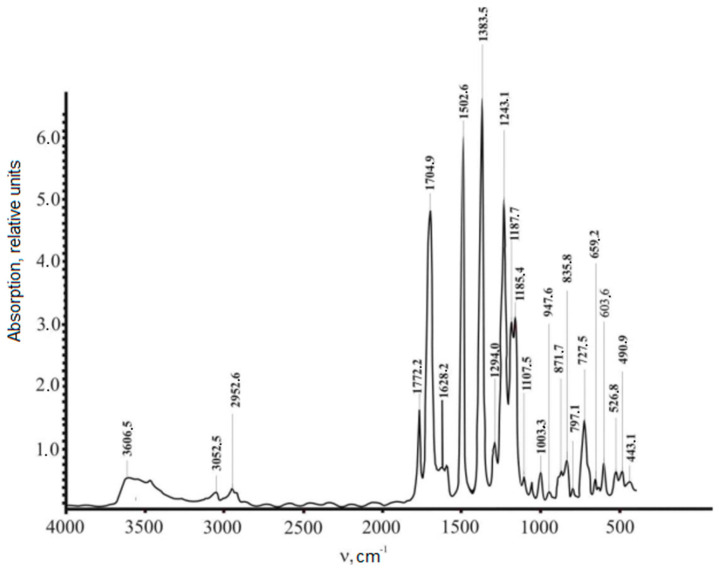
IR spectrum of non-irradiated polyimide film.

**Figure 3:**
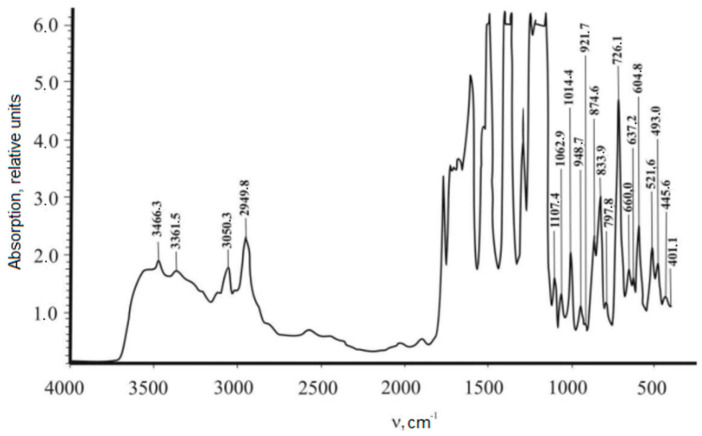
IR spectrum of a polyimide film radiated by electrons with an energy of E = 2 MeV and an absorbed dose of D = 40 MGy.

In this work, the IR spectra of non-irradiated and irradiated polyimide films were measured on a Nicolet 5700 spectrometer, the results of which are shown in Figure 2 and Figure 3. The films were irradiated with electrons with an energy of E = 2 MeV and a dose of D = 40 MGy. The analysis of the spectra of the polyimides was based on a study of the changes in the absorption bands at 720 and 1380 cm^−1^ (the C-N group in the imide cycle) and 1775 cm^−1^ (the C=O group in the imide cycle). In Figure 2 and Figure 3, it can be seen that the radiation caused an increase in the intensity of the indicated spectral lines by a factor of ~2–6 and also significantly increased the bandwidth.

Polyimide composite films with a finely dispersed filler made from a high-temperature superconductor, YBa_2_Cu_3_O_6+x_ (YBCO), were studied. Polyimide (PI) belongs to the class of heat-resistant synthetic polymers containing an imide cyclic group in the structural unit of the macromolecule. A characteristic feature of this polymer is that it has chemical resistance to external aggressive media, as well as high thermal stability at high and low temperatures. [15,16]. Such PCMs have unique properties, combining the advantages of high-temperature superconducting conductors (HTSC) and polymers, namely, these are the structural perfection of the polycrystallites, the mechanical strength and plasticity of polymers, their chemical resistance to aggressive media, etc. [17].

The intensity of the processes of deformation, viscous flow, and destruction of PCM depends strongly on the form of an exponent or a jump in the temperature, pressure, the dose of radiation exposure, or other parameters. The PI-YBCO system has similar properties. These processes are a sequence of elementary actions due to local fluctuations in the thermal energy that are sufficient to overcome the potential energy barrier *Ui*. The mechanism of these elementary actions is based on the fundamental principles of fluctuation dynamics [18,19,20,21,22]. A finely dispersed high-temperature superconductor was chosen as a filler for the PI, namely, YBa_2_Cu_3_O_6+x_, which belongs to the class of layered cuprates. Granulometric analysis of this powder showed that ~75% (wt.) of it consisted of particles ~1 µm in size. The HTSC samples experienced a transition to the superconducting state at Tc = 90 K with a transition width of Δ*T_c_* = 1.5 K. This HTSC material was chosen as the filler in view of the fact that the overwhelming majority of its local states are associated with charged defects. The concentration of defects can be controlled by introducing oxygen atoms. They will manifest themselves in the form of charged centers, which significantly affect the electronic structural state of the PCM.

The homogeneous structure of the composite films was obtained In the following way: the weight of the sample of YBCO was determined on the basis of the selected concentrations of the second component of the compound. A mixture of polyimide lacquer and polyester resin at a given proportion was taken and placed in a three-necked flask, which was filled with m-cresol. An inert gaseous medium was maintained in the flask above the surface of the mixture. This mixture was stirred with uniform heating to 170 °C and a viscous solution was obtained, which was diluted with m-cresol to 7%. Then, the calculated weight of the filler was added to it. The resulting solution was stirred for 2 h at a temperature of 170 °C. The resulting mixture was poured onto a glass substrate and a film of a given size was rolled from it. The resulting films were dried at 100 °C in a muffle furnace [23].

## 3. Results

Exposure of polymeric materials to radiation led to changes in their defective structure, reflecting their physicochemical properties and structure, depending on their original structure, their composition and degree of purity, the characteristics of the incident radiation, the radiation dose, etc. The characteristic course of chemical reactions in polymers under the action of mechanical stresses caused a violation of the degree of ordering of the structure and their level of manifestation. The action of mechanical stresses caused the breaking of chemical bonds (mechano-destruction) in the main chain of the polymers.

Irradiation of polymeric materials caused the formation of radicals as a result of the dissociation of the excited states of macromolecules, as well as the occurrence of secondary reactions of the radicals and ions. These factors affected the concentration of macroradicals formed, reaching up to ~10^19^–10^21^ cm^−3^ [9]. As a result, it caused significant structural changes in these materials, as well as modifications of their properties, which were associated with irreversible processes of structuring and destruction [10]. These processes are opposite in nature and can occur simultaneously. At the same time, the structure of the polymer affected the predominance of one process over another, and also depended on the elements of the components present in the system and the conditions of irradiation [10].

Table 1 shows the informative lines of the IR spectrum of the polyimide film, which lay in the ranges of 500–1100 cm^−1^ and 1700–3500 cm^−1^. In the first interval, the content of the substituted benzene rings of polyimide films was strongly noticeable. At the same time, the content of the associated polyimide groups was notable in the range from 1700 to 3500 cm^−1^. As can be seen, radiation caused an increase in the content of radicals in the samples, which was reflected in an increase in the intensity of the spectrum and was explained by the formation of hydrogen bonds. The change in the intensity of the absorption bands in the polymer (Figure 2 and Figure 3; Table 1) is explained by the fact that irradiation caused competing processes: the formation of cycles involving nitrogen atoms and the formation of nitrogen oxides.

The EPR studies of these films were conducted to determine the operating modes of the equipment that did not cause distortions of the EPR signal shape. Figure 2 presents the measurements of the EPR spectra of one polyimide film irradiated with electrons. The standard mov.ave filter was applied to the sample. The data in Table 1 are given for 15 points for the parameters of the EPR signal (the width ΔH_pp_ and the g-factor).

Table 2 shows that for the same polyimide film sample, the linear width ΔH_pp_ increased by 0.38 mTl when the amplitude of the modulation of the magnetic field changed from 0.6 to 1.6 mTl. This indicates that its modulation broadened. At the same time, this change did not affect the value of the g-factor; it remained constant. Therefore, for serial measurements, a modulation amplitude of 6.15 mTl was chosen, which did not distort the shape of the EPR signal.

Table 3 shows the results of measuring the EPR spectra of polyimide films, both those that were non-irradiated and unbroken (initial) and those radiated with electrons and subjected to stretching to rupture (experimental).

Table 3 shows that the parameters of the EPR signal did not depend on the action of an external load after irradiation. The line width ΔH_pp_ was 0.925 mTl and 0.930 mTl, respectively, and the g-factor is 2.00824 and 2.00791 before and after irradiation.

**Figure 4:**
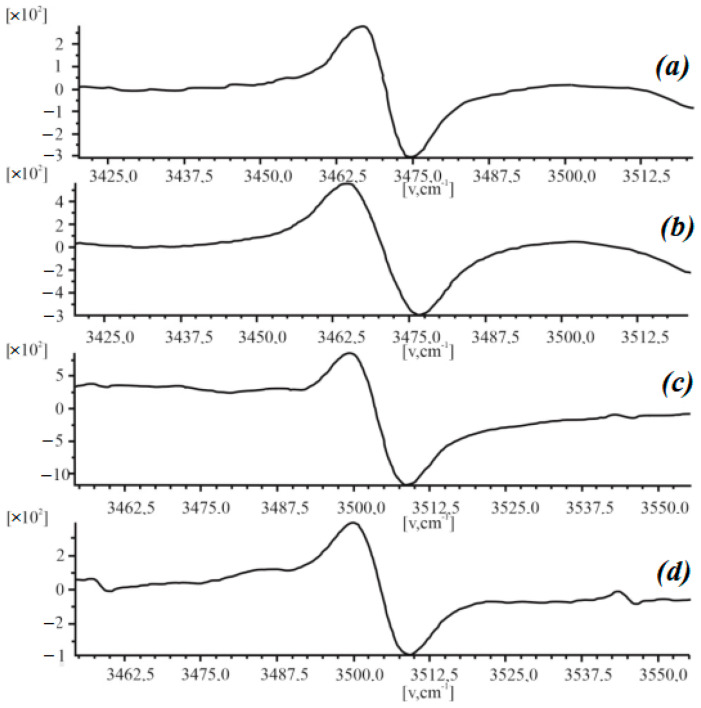
EPR spectra of polyimide films: (**a**) non-irradiated and unbroken; (**b**) non-irradiated and stretched to breaking point; (**c**) radiated with electrons at a dose of 50 kGy and stretched to breaking point; (**d**) radiated with electrons at a dose of 500 kGy and stretched to breaking point.

Figure 4 shows the effects of changes in the intensity of the EPR signal of polyimide films on the concentration of paramagnetic centers. It can be seen that the EPR spectrum was determined by the radicals associated with the associated linear type of the =N-H, –N-H_2_ groups. The concentration of such radicals increased as a result of the action of an external mechanical load until the film broke (Figure 4a,b), which was reflected in an increase in the amplitude of the EPR signal from 3 × 10^3^ to 5 × 10^3^. The relative elongation of the films at the breaking point did not exceed 6.

The impact of electron irradiation on polyimide films followed by their subjection to a mechanical load caused a shift in the spectrum line from 3475.0 cm^−1^ to 3512.5 cm^−1^, with a simultaneous decrease in the signal’s amplitude from 6·× 10^3^ to 4 × 10^3^ (Figure 4c,d). This indicated a decrease in the concentration of radicals of the =N-H, –N-H_2_ groups until their complete disappearance and the formation of new ones as a result of electron irradiation. Figure 4 shows the effects of the action of an external load and the electron irradiation of polyimide films on changes in the EPR spectrum, reflecting the transition of radicals from their original form to their complete disappearance, then to newly formed ones.

A feature of YBa_2_Cu_3_O_6+x_ is that in Positions O4 and O5, the content of labile oxygen can vary within the widest possible range from x = 0 to x = 1. At x = 1, an orthorhombic structure of an O-I compound is formed in the material in the form of a metal phase, such as a superconductor with a superconducting temperature transition Tc of ~90 K. This state is characterized by the fact that, along the b axis, the oxygen position O4 in the …−Cu1−O4−Cu1−… chains are almost all filled, and along the a-axis, the O5 positions in the chains …−Cu1−O5−Cu1… are almost all vacant. At x = 0, YBCO has a tetragonal structure T. It is an insulator. This state of the material is characterized by the fact that the oxygen positions O4 and O5 are vacant, including the orthorhombic O-II phase (x~6.5) with a superconducting transition temperature Tc of ~60 K.

The study of the effects of low-temperature annealing and gamma irradiation, as well as the concentration of filler, on the modification of the macromolecules of polyimide films was carried out by comparing the intensities of the IR and Raman spectra before and after exposure to these physical factors. The choice of the IR spectrometry method was based on the fact that the IR spectra are sensitive to the nature of chemical bonds in polymeric materials, as well as in inorganic crystal lattices and clusters [24].

Therefore, the study of the IR spectra made it possible to obtain information both on the dynamics of the crystal lattice and on the electronic properties of the objects of the study. The method of Raman spectrometry was chosen because it complements the original method. In IR spectroscopy, the incident radiation is absorbed by the sample, due to which, a change in the dipole moment is observed. In Raman spectrometry, the sample itself is the source of radiation. Therefore, in Raman spectrometry, a change in the polarizability of the bond can be observed. Spectral studies by Raman spectroscopy were performed at room temperature on an NT-MDT NTEGRA Spectra spectrometer in the inverted configuration.

The radiation source was a laser with a wavelength of 630 nm. The materials were installed and fixed on an optical substrate. Scanning was performed by the accumulation method to reduce the noise level, with an exposure time of 30 and 100 s, and a period of 1 s. The number of scanning points along each axis was 600 × 600. The IR spectra were recorded by a standard technique at room temperature on a Jasco IR-810 spectrophotometer (Japan) in the wavenumber range of 400–4000 cm^−1^, with ranges of 4200–1200 cm^−1^ and 1400–400 cm^−1^. Samples exposed to air at room temperature were irradiated on an RCM-γ-20 two fixed integral doses of 100 and 600 kGy. The exposure of the source to a dose rate of ^60^Co at a distance of 1 m was 0.16 rad/s. The samples were made in the form of parallelepipedal films. In their working part they had a length of 50 mm, a width of 5 mm, and a thickness of 130 µm.

## 4. Discussion

A feature of the processes of changing the mechanical properties of polyimide films is that as a result of the action of an external mechanical load and electron irradiation, the transition of radicals in these materials from their original form to others occurred, which was accompanied by the complete disappearance of radicals of the first type and the formation of new ones. Such changes in the structure of the polymer were automatically reflected in its mechanical properties. At the same time, the number of radicals in a polyimide film primarily depended on the impact of an external mechanical load and only then on the magnitude of the radiation dose.

**Figure 5:**
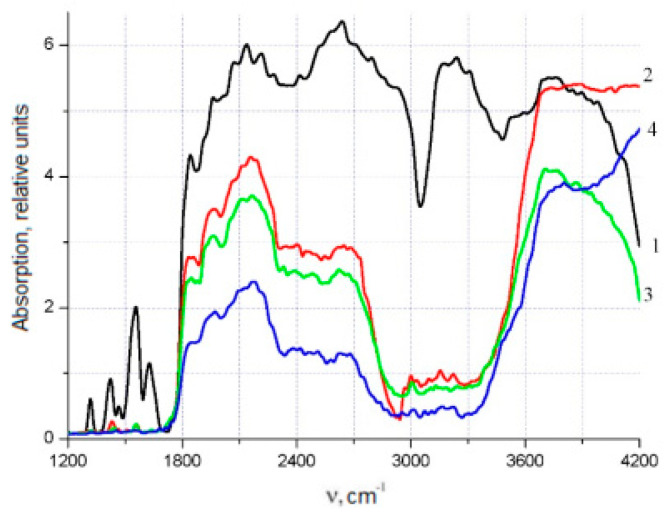
Influence of the concentration of the YBa_2_Cu_3_O_6+x_ filler on the Infrared spectra of polyimide. 1, polyimide; 2, C = 0.05 wt.%; 3, C = 0.1%; 4, C = 0.5 wt.%.

**Figure 6:**
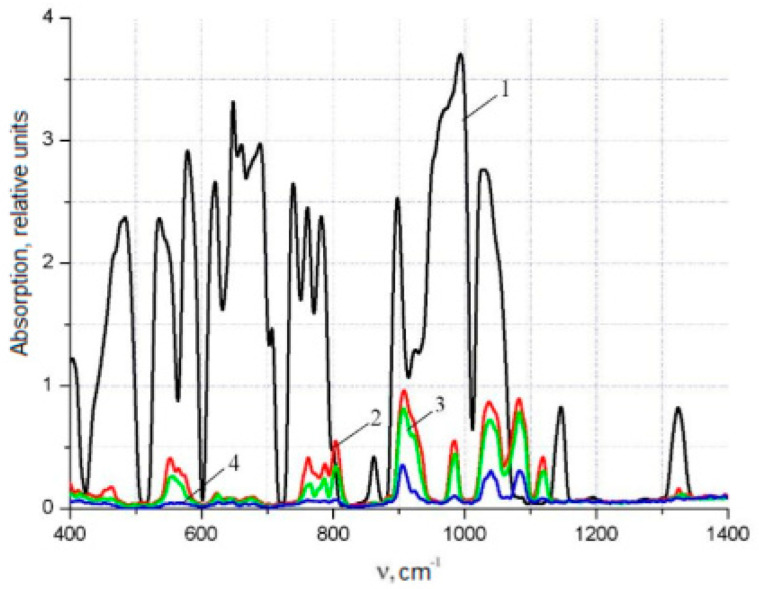
Influence of the concentration of the YBa_2_Cu_3_O_6+x_ filler on the Infrared spectra of polyimide. 1, polyimide; 2, C = 0.05%; 3, C = 0.1%; 4, C = 0.5 wt.%.

The spatial arrangement of the individual structural elements of the polymer is formed by a specific supramolecular arrangement, which cooperates with its internal structure, on the basis of which, its physical properties are formed. To confirm the results of the IR-spectroscopy studies and the structural analysis shown in Figure 5 and Figure 6 and Table 1, we compare the results with those of British scientists’ studies. They investigated PI films’ physical and mechanical properties [15,16]. As a result, we supposed that the electrons’ radiation was significantly reflected in the IR spectrum and expanded the bandwidths 2–6 times. Such changes in the density of polyimide films were due to the formation of hydrogen bonds that occurred under the action of X-rays. Changes in the intensity of the absorption band caused the occurrence of excitation processes, namely, the creation of cycles with the release of nitrogen and the formation of nitrogen oxides.

The elemental analysis showed that the electron irradiation of polyimide films led to the carbon and hydrogen content decreasing from 69.36 wt.% to 68.32 wt.% and from 27.15 wt.% to 28.53 wt.%, respectively. Hence, it follows that the radiation resistance of polyimides substantially depends on the presence of molecular oxygen dissolved in them and the rate of its entry from the environment. An increase in the oxygen content slowed down or completely suppressed the formation of a spatial network of the polymer and led to a decrease in the tensile strength of the material. This indicates that electron irradiation can improve the physicochemical properties of polyimide to a certain extent.

The practice of using irradiation as a technological method makes it possible to carry out a directed change in the properties of a polymeric material, giving it the required technical characteristics. However, it should be taken into account that for a certain group of polymers, radiation exposure worsens their physicochemical properties. The study of the behavior of polymeric materials under extreme conditions, such as ionizing radiation, high- and low-temperature fields, mechanical loads, and other external influences, is a step in developing radiation technologies for obtaining polymeric materials with improved physical, chemical, and mechanical properties.

The irradiation leads to breaks in the bonds of polyimide macromolecules and radicals formation. The number of radicals is reflected in IR spectra intensity. Growth in the number of radicals causes an increase in the intensity of the IR spectrum. Uniaxial stretching and the rupture of non-irradiated polyimide films cause changes in the structure of polyimide macromolecules in the form of an increase in the number of formed radicals, which are closely related to the amplitude of the EPR signal, namely, to its raising. The influence of the concentration of the fine filler YBa_2_Cu_3_O_6+x_ on the modification of polyimide macromolecules was assessed by the change in the intensity of the IR spectra of polyimide at room temperature, as presented in Figure 5 and Figure 6. The spectrum of the polyimide film (Curve 1 in Figure 5 and Figure 6) contained maxima at 1324, 1421, 1555, 2133, 2634, 3238, and 3304 cm^−1^, and a wide absorption band in the range of 2900–3170 cm^−1^. These peaks in their configuration refer to -*CH* connections such as RCH = CHR, R_2_C = CH_2_, and R–CH = CH_2_, as well as to -C-O bonds, which belong to the secondary alcohol groups. In their configuration, some of the spectral peaks correspond to the -C-N and >N-H bonds contained in amino acids and the associated groups of >N-H, -NH_2_ bonds, as well as the >C=C< bonds related to aromatic groups, the -C ≡ C- bonds related to alkynes, and the carboxyl groups formed by -OH bonds.

It can be seen from Figure 5 and Figure 6 (Curves 2, 3, and 4) that the introduction of an HTSC filler caused a significant decrease in the intensity of the initial IR spectrum of the matrix, the maximum value of which was 20.6%. It was established that in the range of wavenumbers 400–1400 cm^−1^ in Figure 6, the intensities of the PCM spectra with YBCO concentrations of 0.05% and 0.1 wt.% (Curves 2 and 3) decreased significantly and did not differ significantly from each other. An increase in the concentration of the filler to 0.5 wt.% led to a significant decrease in the intensity of the spectrum (Curve 4) in the range of 400–1400 cm^−1^. In the range of 1100–1400 cm^−1^, the characteristic peaks of alkynes and the associated groups of =N-H, –NH_2_ practically became zero. It is characteristic that all samples with different filler concentrations had a wide absorption band in the range of 2850–3350 cm^−1^, which is absent in polyimide films (Figure 5). Its formation was affected by the presence of Cu, Y, and Ba crystalline hydrates in the polymer matrix introduced by the filler. The same was observed in [17].

**Figure 7:**
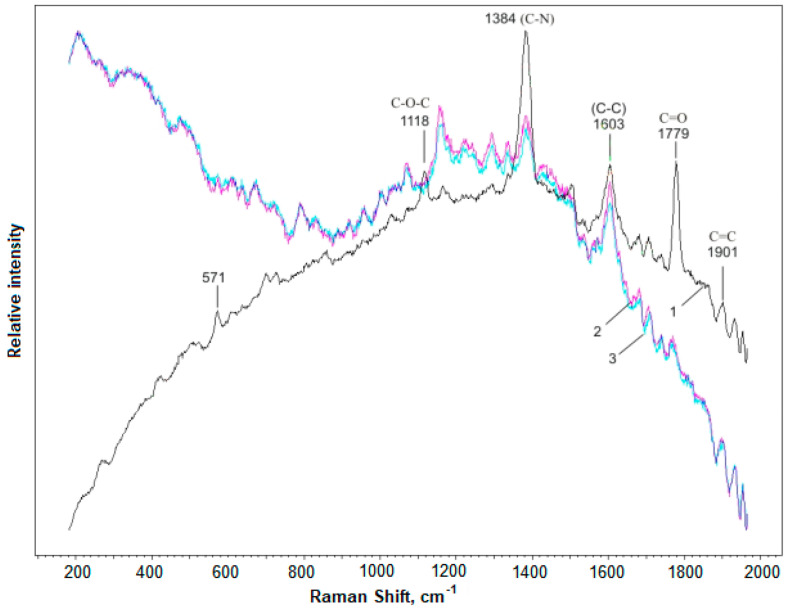
Changes in the Raman spectrum of polyimide composite films with changes in the concentration of the filler: 1, polyimide; 2, with HTSC filler (C = 0.1 wt.%); 3, with HTSC filler (C = 0.5 wt.%).

Figure 6 shows that for polyimide with YBCO filler concentrations of C = 0.05% and C = 0.1 wt.%, the almost-aligned peaks of the polyimide material were partially preserved in the range from 484 to 862, 992, 1147, and 1326 cm^−1^ due to the substituted benzene rings. Increasing the concentration to 0.5 wt.% led to their complete disappearance. This indicated the destruction of bonds in the polyimide macromolecules by the transformation of the structure. Starting from 0.5 wt.%, these bonds disappeared from the macromolecules. The effect of the concentration of the filler on the polyimide was also studied by Raman spectrometry at room temperature, as shown in Figure 7. The introduction of a filler into the HTSC polyimide led to the appearance of new spectral lines in the region of 300–500 cm^−1^, which were inherent in YBCO (see Figure 7). Spectral lines in the region of wavenumbers 300–350 cm^−1^ corresponded to YBCO’s deformation vibrations, which were in good agreement with the results of [19]. Peaks at 450–470 cm^−1^ were characteristic of the oxygen ions O^2+^ and O^3−^ in YBCO. The introduction of a filler concentration of 0.1 wt.% produced a strong change in the Raman spectrum. The intensities of spectral lines decreased to ~50% in the region of 1880–1900 cm^−1^ (C=C), by ~45% cm^−1^ at 1760–1780 cm^−1^ (C=O), and by ~18% at 1380 cm^−1^ (C-N); at 570 cm^−1^, there was an increase of ~55%. This indicated a modification of the structure of the polyimide macromolecules due to the destruction of the bonds between them. Increasing the concentration of filler to 0.5 wt.% did not cause significant changes in the Raman spectrum compared to 0.1 wt.%.

With the introduction of an HTSC filler, the vibration amplitudes of C-N chains at 1380 cm^−1^ and C=O at 1760–1780 cm^−1^ decreased significantly by up to ~20% (see Figure 3). This suggests that the oxygen atoms of the matrix in these bonds are actively involved in the interaction with the particles of the filler, forming a boundary layer between the matrix and the filler. The existence of an active interaction between the matrix’s macromolecules and the particles of HTSC filler was confirmed by observing an increase in the reduced viscosity of polyimide with the introduction of metal oxides of the second group, as shown in Table 4. This indicated that the metal oxides of the second group had a catalytic effect on the course of the polyimide’s imidization reaction by the mechanism of basic catalysis. A solution of polyimide was obtained in m-cresol with a concentration of 0.5 dL/g, and reduced viscosity η_пp_. was determined on an Ubellode viscometer at 20 °C.

These changes in the IR and Raman spectra indicated the modification of polyimide macromolecules and the formation of a boundary layer between the matrix and the particles of filler. The structural activity of the dispersed filler with respect to the macromolecules of the matrix caused the formation of a surface layer of the polymer with special properties around the introduced particles. This was observed in [18]. This layer generally has a significant effect on the properties of composite materials.

The oxygen content in the structure of polyimide films significantly affects their mechanical properties, which was noted in [19]. In [20], the influence of the chemical structure of the polymer binder on the changes in the physicochemical properties of the compositions was revealed. This established the effect of oxygen in the matrix and the conditions for the formation of polymer–ceramic compositions based on YBa_2_Cu_3_O_6,97_ ceramic powder on changes in the physicochemical properties of the compositions. This effect is due to the competing action of two parallel processes. The first process is due to the interaction of individual elements or fragments of polymeric binder macromolecules with the surface of the ceramic grains up to their intercalation into the interlayer space of ceramics. The second is a thermal-oxidative process, with the destruction of the polymer matrix during molding. The deterioration in the plastic properties of PCM samples with YBCO fillers revealed in this work was associated with a high proportion of oxygen in the matrix. A comparative analysis of the Raman and IR spectra of the original polyimide and the composite material presented in Figure 5, Figure 6 and Figure 7 was made.

The Raman spectra (Figure 7) showed that with the introduction of a filler into the HTSC polyimide, new spectral lines formed and strong changes in the intensity of these lines occurred. This was confirmed by the results of [21,22]. In the work of [21,22], it was shown that the emerging peaks at 450–470 cm^−1^ corresponded to the oxygen ions in YBCO. In the material YBa_2_Cu_3_O_6+x_, there is an abnormally high oxidizing pair of Cu^3+^/Cu^2+^. During the formation of the composite, at elevated temperatures, the effect of this pair is enhanced [21]. In [22], the implementation of such an interaction in the complexes was proved. Therefore, the appearance of peaks at 450–470 cm^−1^ in the Raman spectra of polyimide composite materials was due to the interaction of these pairs located on the surface of films of HTSC crystals, with oxygen ions taken from the polymer matrix.

Starting from a filler concentration of 0.1 wt.%, in parallel with changes in the intensity of the spectral lines inherent in polyimide, we observed the appearance of new lines in the Raman spectra. Strong effects were observed in the form of a decrease in the intensity of the spectral lines by ~50% in the range of 1880–1900 cm^−1^ (C=C), by ~45% in the range of 1760–1780 cm^−1^ (C=O), and by ~18% in the range of 1380 cm^−1^ (C–N); at 570 cm^−1^, the intensity increased by ~55%. An increase in the concentration of YBCO in PCM to 0.5 wt.% did not cause significant changes in the nature of the Raman spectra compared with a concentration of 0.1 wt.% (see Figure 7). From the above, we can conclude that a significant effect of the concentration of finely dispersed YBCO on the modification of the structure of macromolecules of polyimide films was achieved with small values up to ~0.1 wt.%.

## 5. Conclusions

1. The obtained experimental results allowed us to conclude that the electron radiation of polyimide caused an increase in the intensity of the IR spectra by a factor of ~2–6 and significantly increased the width of its bands. The most informative lines of the spectrum lie in the intervals of 500–1100 cm^−1^ and 1700–3500 cm^−1^;
2. In the range of 500–1100 cm^−1^, an increase in the intensity of the IR spectra indicated the substitution of benzene rings in the PI films. The interval from 1700 to 3500 cm^−1^ showed the content of the associated groups of macromolecules. An increase in intensity indicated an increase in the content of radicals as a result of irradiation and the formation of hydrogen bonds. The change in the intensity of the absorption bands was caused by the occurrence of competing processes: the formation of cycles involving nitrogen atoms and the formation of nitrogen oxides;
3. Uniaxial stretching to rupture of non-irradiated polyimide films led to an increase in the amplitude of the EPR signal from 3 × 10^3^ to 5 × 10^3^, caused by an increase in the number of radicals;
4. The electron radiation of polyimide films and their subsequent subjection to a mechanical load caused a shift in the spectral line of EPR from 3475.0 cm^−1^ to 3512.5 cm^−1^, with a simultaneous decrease in the signal’s amplitude from 6 × 10^3^ to 4 × 10^3^. This was caused by a decrease in the concentration of radicals of the =N-H and –N-H_2_ groups until their complete disappearance and the formation of new ones. Changes in the EPR spectrum reflected the transition of radicals in polyimide films from one type to another, caused by the action of uniaxial tension and electron radiation;
5. By taking the peculiarities of the relationship between the mechanical properties and the behavior of the structural elements of polymeric materials into account, under the influence of the external physical factors above, it was possible to predict the creation of new ones and improve existing ones;
6. Analyses of the above experimental data of the polyimide–YBa_2_Cu_3_O_6+x_ complex allowed us to draw the following conclusions:
  1. Concentrations of the HTSC filler up to ~0.1 wt.% had a significant effect on the structure of the macromolecules of polyimide films, causing their modification. Changes in the Raman spectra were caused. Spectral lines appeared that are characteristic of YBCO (300–400 cm^−1^), with the intensities of the lines corresponding to oxygen ions increase (470–480 cm^−1^), and there were also maxima in the range of 570–800 cm^−1^, which probably corresponded to the formation of a boundary layer region between the particles of the filler and the polyimide matrix;
  2. A filler concentration of 0.5 wt.% did not cause significant changes in the nature of the Raman spectra compared with 0.1 wt.%. An increase in the concentration of the filler (above 0.1 wt.%) caused a clear manifestation of the absorption band characteristic of YBCO (2850–3350 cm^−1^) due to Cu, Y, and Ba crystalline hydrates in the polymer matrix.
7. The main potential application of the research results lies in improving the reliability of instruments and devices operating in radiation fields and increasing their radiation resistance by irradiation and thermal annealing without changing their chemical structure.

## Figures and Tables

**Table 1:** Comparison of the intensity of the Infrared spectra of a non-irradiated polyimide film and a film radiated with electrons with an energy of E = 2 MeV and a dose of D = 40 MGy.

Chemical Compounds	Frequency Area, cm^−1^	Intensity Increase (Times)
Overtones of PI benzene rings	443.1	2
	490.9	2.8
	526.8	3.6
	603.6	3.4
\	660.0	3.5
Aliphatic groups	726.0	3.3
Substituted PI benzene rings	797.1	3.3
	835.8	3.7
	871.7	3.7
	947.6	4.0
Lactone groups	1772.2	2.5
Associated carboxyl groups of PI	3052.5	6.7
Associated groups =N-H, –N-H_2_ PI	3361.5 and 3466.3	3.8

**Table 2:** Parameters of the electron paramagnetic resonance signal of the polyimide film (width ΔH_pp_ and g-factor).

Sample Name	Sample Mass, Mg	Power, mWt	Amplitude of Modulation, mTl	Width ΔH_pp,_ mTl	g-Factor
Experimental	46	3.99	0.6	0.82	2.00788
Experimental	46	3.99	1.60	1.2	2.00790

**Table 3:** Effect of electron radiation and stretching to rupture on the electron paramagnetic resonance signal parameters of polyimide films.

Sample Name	Sample Mass, Mg	Power, mWt	Amplitude of Modulation, mTl	Width ΔH_pp,_ mTl	g-Factor	
Initial	43	3.99	6.15	0.925	2.00824	
Experimental	26.3	3.99	6.15	0.930	2.00791	

**Table 4:** Characteristics of the reduced viscosity of polyimide in the presence of various additives of elements of the second group.

Type of Additives	η_пp_, 0.5%, dL/g PI_20°C_
-	0.70
MgO	0.88
CaO	1.22
BaO	0.67
CuO	0.95
PbO	0.70
ZnO	0.85

## Data Availability

The data presented in this study are available on request from the corresponding author.
